# Effects of growth years on the quality of cultivated *Bupleurum scorzonerifolium* roots, with wild *Bupleurum scorzonerifolium* roots as a reference

**DOI:** 10.3389/fpls.2025.1552429

**Published:** 2025-03-31

**Authors:** Changsheng Yuan, Feng Zhou, Yihan Wang, Huaizhu Li, Yongliang Li, Shan Guan, Huaizhong Gao, Shikong Gao, Luqi Huang, Yanmeng Liu, Zhilai Zhan

**Affiliations:** ^1^ State Key Laboratory for Quality Ensurance and Sustainable Use of Dao-di Herbs, National Resource Center for Chinese Materia Medica, China Academy of Chinese Medica Sciences, Beijing, China; ^2^ School of Chemistry and Chemical Engineering, Xianyang Normal University, Xianyang, China; ^3^ Shaanxi Health Guardian Liuwei Pharmaceutical Co., Ltd., Yulin, China; ^4^ NiuNiuShun Planting Farmers’ Professional Cooperative in Yuyang District, Yulin, China; ^5^ Shenmu Animal Husbandry Development Center, Yulin, China

**Keywords:** *Bupleurum scorzonerifolium*, growth years, chemical composition differences, transcriptomics and metabolomics, quality difference, molecular mechanisms

## Abstract

**Introduction:**

With the rapid decline in wild Bupleurum scorzonerifolium (BSW) resources, large-scale planting of cultivated B. scorzonerifolium (BSC) has begun. However, the effects of growth years on the quality of BSC, as well as the quality difference between BSC and BSW remain unclear.

**Methods:**

This study utilized trait characteristics, microstructure, cell wall components, sugar components, main pharmacological substances, metabolomics, and transcriptomics as key indicators and techniques, to comprehensively characterize and investigate the molecular mechanisms underlying the quality differences between BSC of different growth ages (1–3 years) and between BSC and BSW.

**Results and discussion:**

As the growth years increased, the root weight of BSC continuously increased, accompanied by root head expansion. Root length and middle and lower section diameters showed significant increases from 1 to 2 years, stabilizing from 2 to 3 years. The ratio of phloem width to xylem radius, along with the contents of total saponins and saikosaponins a, b2, c, and d, exhibited a trend of initial decrease, followed by an increase. Additionally, the number and size of oil cavities and ducts, as well as the volatile oil content, continuously increased. Metabolomic and transcriptomic analyses identified saponins and terpenoids as the primary differential components between different BSC growth years, with genes Bchns02G071080 and Bchns05G031860 (saponins) and Bchns01G03800 (terpenoids) identified as potential key regulatory genes. Furthermore, compared to the long-established, high-quality BSW, 3-year-old BSC (BSC3) showed the closest resemblance in morphological traits, microstructure, and chemical component content, particularly for key pharmacological substances, such as volatile oil, total saponins, and saikosaponins a, b2, c, and d. Considering both quality and economic benefits, BSC3 is recommended as a substitute for BSW. To the best of our knowledge, this is the first study to report the effects of growth years on the quality of BSC, offering valuable insights for guiding the artificial cultivation and quality assurance of B. scorzonerifolium.

## 1 Introduction

*Bupleurum scorzonerifolium* Willd. belongs to the genus *Bupleurum*, family Umbelliferae (POWO, 2025). It’s a medicinal source of *Bupleuri Radix* (syn. *Radix Bupleuri*, Chaihu in Chinese) (Chinese Pharmacopoeia Commission, 2020). Its root has been used for medicinal purposes in China for over a thousand years and is officially listed as a medicinal substance in pharmacopeias in China, the United Kingdom, Europe, and South Korea (Sun et al., 2020). Traditionally, it has been employed to treat fever (Chen et al., 2022; Kang et al., 2023) and emotional disorders (Wang and Peng, 2020; Mu and Ma, 2023). The main active constituents in *B. scorzonerifolium* root include saponins, volatile oils, and polysaccharides (Jiang et al., 2020), which exhibit pharmacological activities, such as anti-inflammatory, antidepressant, antitumor, immune-modulating, neuro-regulatory, and hepatoprotective effects (Li et al., 2007; Ashour and Wink, 2011; Mu and Ma, 2023; Teng et al., 2023).

The quality differences between cultivated *B. scorzonerifolium* (BSC) and wild *B. scorzonerifolium* (BSW), as well as the optimal growth years for BSC, are pressing issues in the production and use of *B. scorzonerifolium* root. Wild medicinal plants are vital sources of traditional Chinese medicine (TCM). Compared to cultivated varieties, wild medicinal materials are highly valued in the market because of their long medicinal history, superior appearance, and greater clinical efficacy (Wang et al., 2024a). Historically, *B. scorzonerifolium* root was harvested from wild plants, with Yulin City in Shaanxi Province recognized as the authentic production area (Zhao et al., 2020). However, modern market demands have outstripped the supply of wild resources. In response, large-scale cultivation of *B. scorzonerifolium* has been established in the authentic production area. Despite this, quality differences between BSC and BSW remain unresolved. Similarly, the quality of TCM is closely tied to its growth year (Chen et al., 2021), as demonstrated in ginseng (Zhang et al., 2012; Cui et al., 2015), dendrobium (Liu et al., 2018), and astragalus (Wang et al., 2024). However, the quality differences among BSCs of different growth years are yet to be determined. While previous studies have examined the quality differences and molecular mechanisms of *B. scorzonerifolium* from different regions (Wang et al., 2023) and developmental stages (Yu et al., 2023), no research has been conducted on these aspects between BSC of different growth years.

The essence of TCM quality evaluation lies in the integration of multiple indicators, including traditional sensory evaluation, microscopic characteristics, chemical composition, and biological effects, with clinical efficacy at its core (Wang et al., 2021). Traditional sensory evaluation indicators for TCM encompass overall appearance, cross-sectional features, and texture. These indicators not only provide intuitive insights into quality but also correlate significantly with the content of chemical substances, especially bioactive compounds. For example, ginseng with a long stipe, multiple rootlets, and a spongy texture is associated with higher total saponin content (Dai et al., 2020). Similarly, turmeric with a golden-yellow cross-section is positively correlated with active compounds such as curcumin (Wang et al., 2018). Microscopic features also strongly relate to the content of bioactive compounds (Fan et al., 2019). These bioactive compounds serve as indicators of biological effects, clinical efficacy, and safety profiles (Long et al., 2021). Notably, metabolomics and transcriptomics are gaining prominence in elucidating the molecular mechanisms underlying the formation of superior TCM traits and the accumulation of pharmacologically active components (Tian et al., 2022).

Based on this, the present study systematically compared BSC of different growth years from the perspectives of macroscopic traits, microscopic characteristics, cell wall components, carbohydrate components, main pharmacological substances, metabolomics, and transcriptomics, taking BSW as a quality standard. This study elucidated the differences and relationships at various levels, exploring the underlying mechanisms of quality variation. The findings provide a foundation for the high-quality production of *B. scorzonerifolium*.

## 2 Materials and methods

### 2.1 Plant materials, chemicals, and reagents

From late August to early September 2023, *B. scorzonerifolium* cultivated for 1 year (BSC1), 2 years (BSC2), and 3 years (BSC3), and BSW were collected in Yulin City, Shaanxi Province. All BSC samples were grown by the NiuNiuShun Planting Farmers’ Professional Cooperative in Yuyang District, Yulin City, and Shaanxi HealthGuardian Liuwei Pharmaceutical Co., Ltd. The growing environment and cultivation practices for all cultivated samples were consistent. All cultivated *B. scorzonerifolium* were seeded from wild *B. scorzonerifolium*. The raw land was cultivated for direct seeding and no agronomic management practices were carried out until harvesting. The BSW samples were collected from mountainous areas (38°26′N, 110°0′E) near the cultivated regions. The collection sites were located at an altitude of 1,280–1,310 m, and belongs to the hilly and gully area of the Loess Plateau. The terrain is sunny and gently sloping (slope 10–15°). The soil is yellow loamy soil. After collection, the roots were washed with purified water and dried with absorbent paper. All roots were randomly divided into three groups. One group was stored at -80°C for transcriptomic and metabolomic analysis. It included three biological replicates, with each replicate comprising at least five individual roots. One group was prepared for microscopic observations. One group was naturally air-dried for trait observation, then ground into powder using a Mixer Mill MM500 Vario (Retsch, Germany), and stored for the determination of hemicellulose, cellulose, lignin, starch, total polysaccharides, total saponins, volatile oils, and saikosaponins a, b2, c, and d.

Glucose; saikosaponins a, b2, c, and d; and other substances used in this study were of > 98% purity and purchased from Beijing Beite Renkang Biopharmaceutical Technology Co., Ltd. All reagents were of analytical grade.

### 2.2 Macroscopic traits and microscopic observation

An electronic camera (Canon EOS-800D, Lens EF 24-105 mm f/4 L IS USM, Macro Lens EF 100 mm f/2.8 L IS USM, Japan) was used to capture images of the whole, local, and cross-sectional characteristics of *B. scorzonerifolium* roots (Wang et al., 2024b). Root length, weight, and diameters of the upper, middle, and lower sections were measured, with at least 30 roots measured per group. Differences in traits among the groups were compared and described.

A fresh section approximately 7 mm thick was cut from the middle of a 1 cm root segment located below the head of BSR. After dehydration and fixation using ethanol solutions and paraffin, samples were processed into permanent sections (10–15 µm) for safranin-fast green staining using a cryostat (Leica CM1860, Germany) at -22°C. Stained sections were mounted with resin and observed under an optical microscope (Olympus BX51, Japan) to analyze and compare the microstructural features of each group (Wang et al., 2024b).

### 2.3 Quantitative analysis of hemicellulose, cellulose, lignin, and starch

The detergent fiber analysis method by Van Soest, as described elsewhere (Liu et al., 2013), was applied. Sequential treatments using neutral detergent, acid detergent, 72% sulfuric acid, and ash residue procedures were conducted using a fiber analyzer (ANKOM220, US) to measure hemicellulose, cellulose, and lignin contents. Starch content was determined using the anthrone colorimetric method as described in reference (Chen, 1984).

### 2.4 Quantitative analysis of water-soluble polysaccharides

Anhydrous glucose was dissolved in pure water to prepare a 2.0 mg/mL stock solution and serially diluted to obtain eight gradient reference solutions. Each solution (0.2 mL) was mixed with 0.1 mL of 5% phenol reagent and 0.5 mL of concentrated sulfuric acid, then incubated in a 90°C water bath for 20 min before cooling to room temperature. Absorbance at 492 nm was measured using a Varioskan Flash microplate reader (Thermo, US), and a standard curve was constructed.

Sample measurement followed the method described in reference (Wang H. et al., 2024). Briefly, 0.05 g of powdered sample was placed in a 2 mL centrifuge tube with 0.5 mL of water. After ultrasonic treatment in an SB-800DTD ultrasonic cleaner (Xinzhi Biotechnology, China) for 25 min and centrifugation at 10,000 g for 10 min, the supernatant (0.2 mL) was combined with 0.8 mL of anhydrous ethanol. After standing at 4°C overnight, the samples were centrifuged at 10,000 g for 10 min. The resulting precipitate was dissolved in 1 mL of water to obtain the sample solution. A 0.2 mL aliquot was taken, and the rest of the procedure followed the same steps as the reference solution. The water-soluble polysaccharide content was calculated based on absorbance.

### 2.5 Quantitative analysis of total-saponins

Standard curve preparation: An appropriate amount of saikosaponin a was weighed, and pure methanol was added to prepare a stock solution with a concentration of 6 mg/mL. This stock solution was serially diluted to obtain 11 gradient reference solutions. For each gradient solution, 0.1 mL was taken and evaporated to dryness under a nitrogen stream. Subsequently, 0.2 mL of 5% vanillin-acetic acid solution and 0.8 mL of perchloric acid solution were added. The mixture was incubated in a 60°C water bath for 15 min and then cooled in an ice-water bath to room temperature. After mixing, 0.3 mL of the reaction mixture was taken, and 0.7 mL of glacial acetic acid was added. The mixture was shaken again, and the absorbance at 608 nm was measured using a Varioskan Flash microplate reader. A standard curve was constructed based on the absorbance values of the gradient reference solutions.

Sample measurement: Following the method described in reference (Wang et al., 2024b), 0.05 g of powdered sample was placed in a 2 mL centrifuge tube, and 1.75 mL of pure methanol was added. After thorough mixing, the sample was subjected to ultrasonic treatment in an SB-800DTD ultrasonic cleaner for 30 min. The sample was then centrifuged at 12,000 rpm for 10 min. A 0.1-mL aliquot of the supernatant was carefully transferred, and the subsequent steps followed the same procedure as the reference solution. The total-saponins content in the sample was calculated based on the absorbance value and the standard curve.

### 2.6 Quantitative analysis of volatile oils

A suitable amount of sample powder was weighed, and a sample-to-solvent ratio of 1:25 was prepared by adding an n-hexane solution. The mixture was subjected to ultrasonic treatment at a frequency of 40 kHz for 15 min. After centrifugation at 12,000 rpm for 10 min, a certain volume of the supernatant was collected, evaporated, and concentrated. The volatile oil content was calculated based on the concentration ratio of the extract to the initial sample weight (Zhang et al., 2018).

### 2.7 Quantitative analysis of four saikosaponins

A suitable amount of saikosaponins a, b2, c, and d was weighed, and 1 mL of methanol was added to prepare a mixed standard solution. The solution was then diluted into 13 gradient concentrations, and standard curves were constructed using the peak areas of each standard. A 0.05 g powdered sample was prepared according to the sample preparation method described in section 2.5 and then concentrated 10 times to obtain the sample solutions. Detection was performed using ultra performance liquid chromatography-evaporative light scattering detector (UPLC-ELSD) under the following conditions: chromatographic column, Waters BEH C18 column (1.7 μm, 2.1 mm × 100 mm); mobile phase, 0.1% formic acid in water (A) and 0.1% formic acid in acetonitrile (B). The gradient program was as follows: 0–4 min, 98–80% A; 4–11 min, 80–66% A; 11–13 min, 66–64% A; 13–20 min, 64–60% A; 20–25 min, 60–50% A; 25–28 min, 50–5% A; 28–31 min, 5% A; 31–32 min, 5–98% A; 32–35 min, 98% A. The column temperature was maintained at 40°C, and the flow rate was 0.5 mL/min with a 3 μL injection volume. The ELSD was set with the drift tube temperature at 50°C, gain at 500, and gas pressure at 40 psi with a nebulizer cooling mode. The content of saikosaponins a, b2, c, and d in the samples was calculated based on the peak areas and the corresponding standard curves.

### 2.8 Metabolomics analysis

#### 2.8.1 Sample preparation

All samples were dried using a vacuum freeze dryer and then ground into powder using a Mixer Mill MM500 Vario (Retsch, Germany) at a frequency of 30/s for several minutes. A 0.20 g aliquot of the powder was dissolved in 4 mL of 70% methanol and subjected to ultrasound at a frequency of 40 kHz for 1 h in an SB-800DTD ultrasonic cleaner (China Xinzhi Biotechnology). The mixture was centrifuged at 12,000 rpm for 10 min, and the supernatant was filtered through a 0.22-μm organic filter membrane to obtain the sample solution for UPLC-MS analysis (Wang et al., 2024b).

Additionally, a 0.20 g aliquot of the sample powder was dissolved in 3 mL of n-hexane. The solution underwent the same ultrasonic treatment, centrifugation, and filtration process. The supernatant was concentrated to obtain the sample solution for GC-MS analysis (Zhang et al., 2018). Equal amounts of each sample solution were mixed to prepare the quality control (QC) samples for both UPLC-MS and GC-MS analyses.

#### 2.8.2 Conditions for UPLC-MS and GC-MS analyses

The UPLC-ESI-QTOF-MS system (UPLC, ACQUITY; MS/MS, SYNAPT XS; Waters, USA) equipped with a Waters BEH C18 column (1.7 μm, 2.1 mm × 100 mm) was used for UPLC-MS/MS analysis. The mobile phase consisted of ultrapure water with 0.1% formic acid (A) and acetonitrile with 0.1% formic acid (B). The elution gradient program was as follows: 0 min, 98% A; 0–14 min, 98–69% A; 14–17 min, 69–68.7% A; 17–20 min, 68.7–68% A; 20–21 min, 68–64% A; 21–27 min, 64–60% A; 27–34 min, 60–50% A; 34–38 min, 50–40% A; 38–41 min, 40–2% A; final 2% A maintained for 1 min. The flow rate was 0.5 mL/min, with a column temperature of 40°C and an injection volume of 1 μL.

The GC-MS system (GC, TRACE1310; MS, TSQ8000; Thermo, USA) equipped with an automatic sampler (AI/AS 1310) was used for GC-MS analysis. The system employed a Thermo TG-5MS column (30 m × 0.25 mm × 0.25 μm). The oven temperature program was as follows: 60°C held for 2 min, increased to 140°C at 15°C/min and held for 2 min, then increased to 270°C at 5°C/min and held for 3 min. The split mode was used with a split flow of 10.0 mL/min and a split ratio of 10. The inlet temperature was set to 260°C. The carrier gas flow rate was 1.0 mL/min, and the injection volume was 1 μL.

#### 2.8.3 ESI-QTOF-MS and EI-MS conditions

ESI-QTOF-MS analysis was conducted in negative ion mode, with data acquisition in centroid mode. Instrument parameters were as follows: ion spray voltage, 2,000 V; source temperature, 100°C; desolvation temperature, 450°C; sampling cone, 80; source offset, 30.0; cone gas flow and desolvation gas flow, 50 and 900 L/h, respectively; and ramp trap collision energy, 30–70 eV.

EI-MS analysis was conducted in positive ion mode, with the following instrument parameters: ionization mode, electron impact (EI) at 70 eV; ion source temperature, 250°C; MS transfer line temperature, 280°C; mass scan range, 40–550 m/z. Mass spectrometric signal acquisition began 3 min after program initiation.

#### 2.8.4 Data processing

For UPLC-MS data, qualitative analysis was performed using (1) the internal *Bupleurum* species compound library with primary and secondary mass spectrometry data and (2) standard reference compounds. Quantitative analysis was based on the total ion intensity of identified compounds using TOF-MS. GC-MS metabolites were qualitatively analyzed by deconvoluting raw data with AMDIS software (version 2.73) and comparing results with the NIST2020 database, with a minimum matching coefficient of 80 (Cerdán-Calero et al., 2012).

UPLC-MS data were processed using Progenesis QI software, including peak alignment, extraction, deconvolution, and normalization. GC-MS data were analyzed with Xcalibur software for peak integration and manual correction. Processed data were imported into SIMCA 14.1 for statistical analyses. Principal component analysis (PCA) was used for unsupervised pattern recognition, while partial least squares discriminant analysis (PLS-DA) was conducted for supervised pattern recognition. All variables were Pareto-scaled prior to analysis. A 200-fold six-fold cross-validation test assessed potential model overfitting. Metabolites with the top eight VIP scores identified via PLS-DA were considered significantly different.

### 2.9 Transcriptomic analysis

Total RNA was extracted using the RNAprep Pure Plant Plus Kit (TIANGEN BIOTECH). RNA concentration and integrity were measured using an Agilent 5400 system (Agilent Technologies, CA, USA). A cDNA library was constructed via PCR enrichment, and sequencing was performed on the Illumina Novaseq 6000 PE150 platform. CASAVA base recognition was used to convert sequence data into high-quality raw reads. FASTQ software (version 0.23.1) filtered the raw reads to produce clean reads, which were aligned to the reference genome using Hisat2 (version 2.0.5). The reference genome, provided by a collaborating scientific institution, has not yet been published.

New genes were predicted using StringTie (version 1.3.3b), and gene expression levels were calculated in fragments per kilobase of transcript per million fragments mapped (FPKM). Differentially expressed genes (DEGs) were identified using DESeq2 (version 1.20.0) under the criteria of |log2FC| > 1 and *p* < 0.05.

## 3 Results and discussion

### 3.1 Differences in macroscopic traits

**Figure 1:**
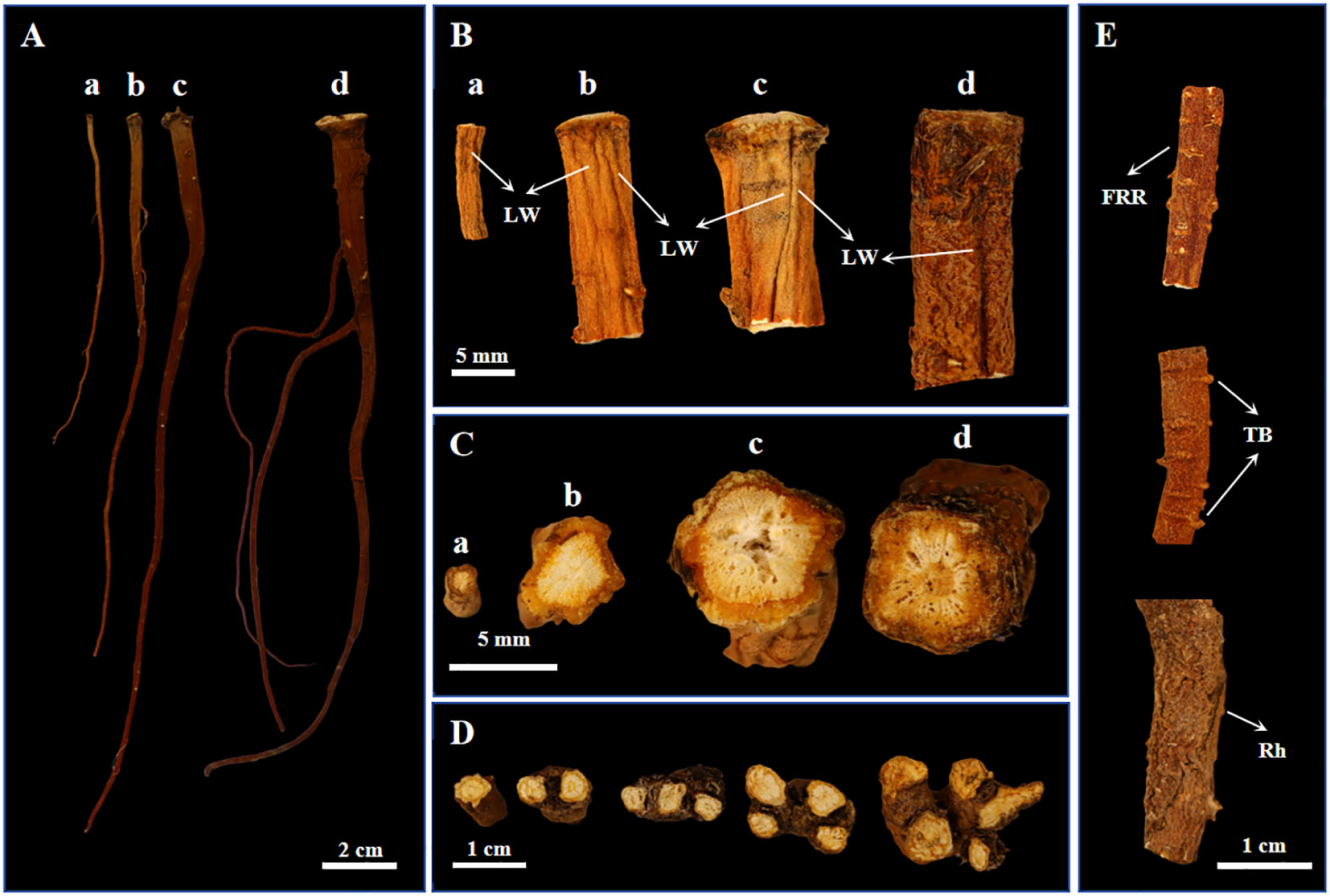
Phenotypic images of cultivated *Bupleurum scorzonerifolium* at 1-3 growth years (BSC1, BSC2, and BSC3) and wild *Bupleurum scorzonerifolium* (BSW). **(A)** Overall image; **(B)** Root segment near the root apex; **(C)** Transverse section; **(D)** Cross-sections of BSW rhizomes containing 1–5 rhizomes (from left to right); **(E)** Fibrous root remnant (FRR), transverse bulges (TB), and rhytidome (Rh) of BSW. Labels: a, BSC1; b, BSC2; c, BSC3; d, BSW; LW, longitudinal wrinkles.

BSC1, BSC2, BSC3, and BSW exhibited similar external characteristics. The roots were conical in shape, with the main root being straight or slightly curved (**Figures 1A**). The apex displayed numerous fine hair-like dead leaf fibers as well as some visible annular striations, while the lower part was mostly unbranched or slightly branched (**Supplementary Figures 1, 2**). The epidermis showed longitudinal wrinkles (**Figures 1B**). The texture was brittle or slightly soft, easily broken, with a slightly flat cross-section that lacked obvious fibrous structures. The root skin was reddish-brown and emitted a rancid oil odor.

However, significant changes in weight, length, and the upper, middle, and lower diameters of cultivated BSC were observed with increasing growth years. First, the root weight significantly increased (**Supplementary Figure 3A**). BSC1 weighed 0.12 ± 0.04 g, while BSC2 and BSC3 weighed 0.66 ± 0.16 g and 0.94 ± 0.65 g, respectively. Second, the main root length significantly increased (**Supplementary Figure 3B**), from 11.43 ± 2.43 cm in the first year to 14.85 ± 2.49 cm in the second year, before slightly decreasing to 13.63 ± 3.13 cm in the third year. Additionally, the head of the root expanded continuously (**Supplementary Figure 3C**), with the diameter increasing from 2.07 ± 0.41 mm in the first year to 4.12 ± 0.71 mm in the second year and 5.62 ± 1.61 mm in the third year. The middle and lower parts of the root thickened significantly from the first to the second year and stabilized between the second and third years (**Supplementary Figures 3D, E**).

In contrast, BSW exhibited a single root weight of 1.03 ± 0.54 g, a length of 13.94 ± 2.74 cm, and upper, middle, and lower diameters of 5.37 ± 1.73 mm, 2.23 ± 0.52 mm, and 1.37 ± 0.45 mm, respectively. Among the BSC samples, only BSC3 showed no significant differences in root weight, length, or upper and middle diameters compared to BSW.

Transverse section analysis revealed distinct xylem characteristics among the samples. BSC1 had a loose xylem, BSC2 had a compact xylem, and BSC3 had a loose xylem with visible fissures. Similarly, the xylem of BSW was loose, with fissures arranged in a radial pattern (**Figure 1C**). Regarding texture, BSC2 was slightly hard, while BSC1, BSC3, and BSW were relatively soft. Notably, BSW typically exhibited 1–5 rhizomes (**Figure 1D**), whereas rhizomes were rarely observed in BSC. Additionally, BSW often showed more pronounced fibrous root remnants, transverse bulges, and rhytidome (**Figure 1E**). These features may serve as distinguishing traits between BSC and BSW.

The root length, diameter, and texture of *B. scorzonerifolium* are closely associated with its quality. According to the Ming Dynasty Chinese material medica *Ben Cao Pin Hui Jing Yao* (Liu, 2019), “*Radix Bupleuri* with long and soft roots is preferred.” The species referred to in this text is identified as *B. scorzonerifolium* (Zhao et al., 2020). Similarly, the modern *Zhong Hua Yao Hai* (Ran, 1993) states: “*B. scorzonerifolium [ … ]* roots that are thick, long, and without rootlets are considered the best.” Furthermore, *500 Commonly Used Traditional Chinese Medicinal Herbs: A Guide to Identification* (Lu, 1999) states: “*B. scorzonerifolium* is best with a single root, soft texture, and a rancid oil odor.”

Modern research confirms that longer and thicker *B. scorzonerifolium* roots generally contain higher levels of volatile oils and saikosaponins within a specific range (Wang et al., 2023). The characteristics of the BSW samples collected in this study align with those of high-quality *B. scorzonerifolium* as described in historical texts, suggesting that these wild samples represent the superior *B. scorzonerifolium* used in ancient times. Based on root weight, root length, upper root diameter, texture, and transverse section characteristics, BSC3 closely resembles BSW phenotypically.

### 3.2 Differences in microstructure

**Figure 2:**
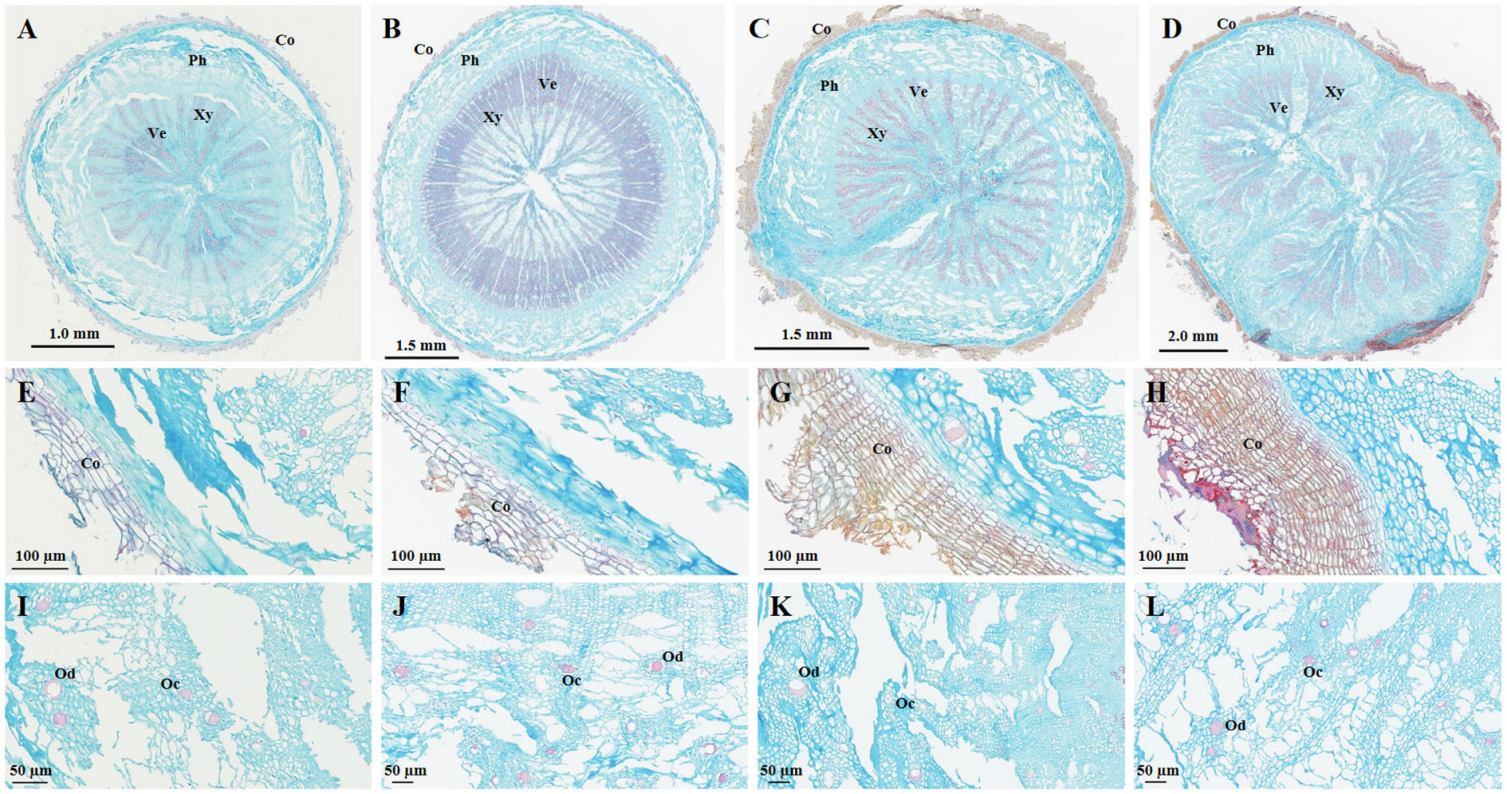
Microscopic images of cultivated *Bupleurum scorzonerifolium* at 1-3 growth years (BSC1, BSC2, and BSC3) and wild *Bupleurum scorzonerifolium* (BSW). **(A–D)** Overall characteristics; **(E–H)** Cork layer; **(I–L)** Oil ducts and cavities. Labels: Co, cork; Ph, phloem; Xy, xylem; Ve, vessel; Od, oil ducts; Oc, oil cavities.

First, regarding the cork layer, the number of cork cells in BSC aged 2–3 years significantly increased, with BSW showing the highest number of cork cells. The cork layer in BSC1 and BSC2 consisted of 3–8 and 3-11 layers of cells respectively (**Figures 2E, F**), while BSC3 exhibited 11–25 layers of cork cells (**Figure 2G**). BSW had 12–30 layers of cork cells, with dense red cork tissue (**Figure 2H**). The cork tissue forms the basis of the periderm and rhytidome, and the differences in cork layer structure directly explain the presence of rhytidome in BSW, which was absent in BSC1, BSC2, and BSC3.

Next, comparing the phloem characteristics, the size and number of phloem fissures, as well as the ratio of phloem width to xylem radius (PXR), BSC showed initially decreasing and then increasing trends over the 1–3 years of growth. BSC1 exhibited large and numerous phloem fissures, with the PXR ranging from 0.79 to 0.82 (**Figure 2A**). In BSC2, the phloem fissures were smaller and fewer, with a PXR ranging from 0.35 to 0.38 (**Figure 2B**). BSC3 displayed smaller but more numerous phloem fissures, with a PXR ranging from 0.57 to 0.73 (**Figure 2C**). BSW had almost no phloem fissures, with a PXR ranging from 0.64 to 0.91 (**Figure 2D**).

Furthermore, BSC1 and BSC2 contained fewer oil ducts and cavities, with most diameters ranging from 10 to 20 µm (**Figures 2I, J**). In contrast, BSC3 and BSW exhibited numerous oil ducts and cavities, with most diameters ranging from 20 to 40 µm, and some reaching up to 80 µm (**Figures 2K, L**).

The phloem is also the main site for the accumulation of saponins and volatile oils (Tan et al., 2008; Cai et al., 2009; Zheng et al., 2009). The number and size of oil ducts and cavities are closely related to the secretion and accumulation of volatile oils (Cai et al., 2008). Generally, a larger PXR, along with more numerous and larger oil ducts and cavities, correlates with higher levels of saponins and volatile oils (Wang et al., 2023). Among the groups, BSC1 had the largest PXR, followed by BSW and BSC3, with BSC2 having the smallest PXR. BSC3 and BSW had more numerous and larger oil ducts and cavities than BSC1 and BSC2. These observations provide microscopic evidence for the significantly higher total saponin content in BSC1, BSC3, and BSW than that in BSC2, as well as the significantly higher volatile oil content in BSC3 and BSW than that in BSC1 and BSC2.

Finally, comparing the morphology and number of vessels, all four *B. scorzonerifolium* samples exhibited xylem vessels arranged in a radial pattern. However, the number and density of xylem vessels were significantly higher in BSC2 than those in BSC1, BSC3, and BSW (**Figures 2A–D**). This serves as microscopic structural evidence for the absence of fissures in the cross-section of BSC2’s xylem and its more compact texture than the other groups.

In summary, significant differences were observed in the cork tissue, phloem, and xylem structures among the BSC samples. As the growth years increased, the size and number of phloem fissures and the PXR initially decreased and then increased, while the number and density of xylem vessels initially increased and then decreased. Additionally, the cork cell number and the number and diameter of oil ducts and cavities showed no significant differences between BSC1 and BSC2 but substantially increased between BSC2 and BSC3. Overall, BSC3 exhibited structural characteristics most similar to those of BSW across these aspects.

### 3.3 Differences in the contents of cell wall components and carbohydrate components

**Figure 3:**
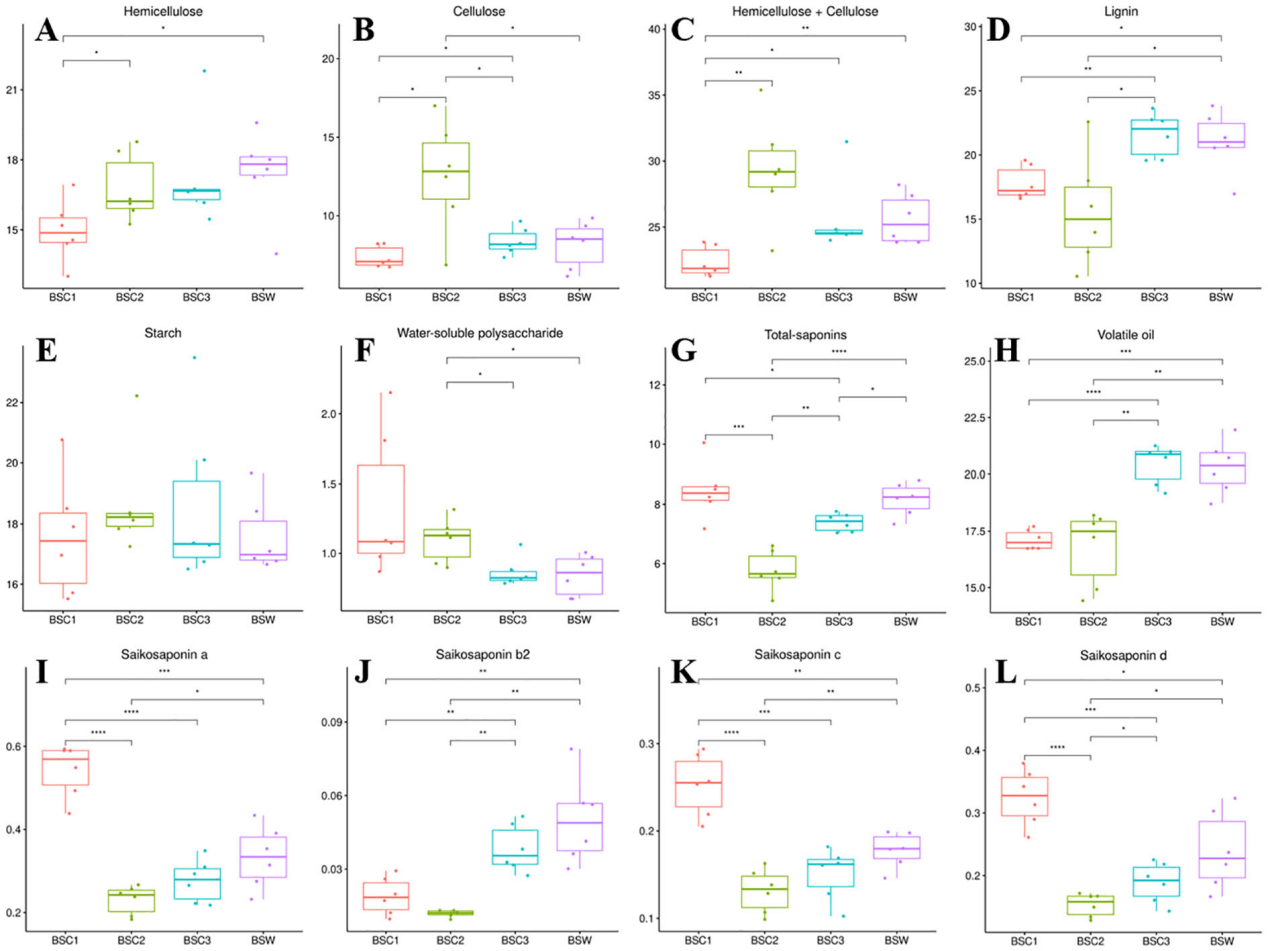
Quantitative analysis of various components of cultivated *Bupleurum scorzonerifolium* at 1-3 growth years (BSC1, BSC2, and BSC3) and wild *Bupleurum scorzonerifolium* (BSW) (%). **(A)** Hemicellulose; **(B)** Cellulose; **(C)** Hemicellulose + Cellulose; **(D)** Lignin; **(E)** Starch; **(F)** Water-soluble polysaccharides; **(G)** Total-saponins; **(H)** Volatile oil; **(I)** Saikosaponin a; **(J)** Saikosaponin b2; **(K)** Saikosaponin c; **(L)** Saikosaponin **(d)** Significance levels: *p < 0.05; **p < 0.01; ***p < 0.001; ****p < 0.0001.

The content of hemicellulose significantly increased between BSC1 and BSC2, with no notable change observed between BSC2 and BSC3 (**Figure 3A**). Cellulose content showed a significant increase (*p* < 0.05) from BSC1 to BSC2, followed by a significant decrease (*p* < 0.05) from BSC2 to BSC3 (**Figure 3B**). The combined content of hemicellulose and cellulose was the highest in BSC2 compared to that in the other groups (**Figure 3C**). In terms of hemicellulose and cellulose content, as well as their total, BSC3 exhibited values most similar to those of BSW.

Hemicellulose, cellulose, and lignin are crucial components of the plant cell wall, playing vital roles in processes, such as elongation and thickening. Cellulose provides strength and elasticity to the plant cell wall, while hemicellulose enhances the wall’s structure through interactions with cellulose (Scheller and Ulvskov, 2010). The high levels of hemicellulose and cellulose in BSC2 are likely chemical factors contributing to its increased hardness, mechanical strength, and the greater number of xylem vessels than those in other groups.

The lignin content showed no significant difference between BSC1 and BSC2 but increased significantly from BSC2 to BSC3. Lignin content in BSC3 was comparable to that of BSW (**Figure 3D**), potentially due to the higher number of cork cells in BSC3 and BSW relative to BSC1 and BSC2.

Starch content remained consistent across BSC1, BSC2, BSC3, and BSW, ranging from 16.98% to 18.23% (**Figure 3E**). Water-soluble polysaccharide content significantly decreased between BSC2 and BSC3, with BSC3 showing levels similar to those of BSW (**Figure 3F**). The methodological details for determining water-soluble polysaccharide content are provided in **Supplementary Table 1**.

### 3.4 Analysis of the differences in the content of major bioactive compounds

Saponins and volatile oils are the primary pharmacological substances in *B. scorzonerifolium*. The total-saponins content was the highest in BSC1, significantly decreasing (*p* < 0.001) from BSC1 to BSC2, followed by a significant increase (*p* < 0.01) from BSC2 to BSC3. BSC1 and BSW exhibited similar total-saponins levels, with no significant differences (**Figure 3G**). A similar trend has been reported in *B. chinense*, where the total-saponins content was higher in younger plants (≤ 1 year) than that in perennials (≥ 2 years) [35]. Integrated analysis of the microscopic structure (Section 3.2) and quantitative cell wall composition (Section 3.3) suggests that BSC1, at its early growth stage, exhibits thinner cork layers and lower levels of structural/defensive cell wall components (e.g., hemicellulose, cellulose), resulting in weaker physical stress resistance. Consequently, BSC1 prioritizes the synthesis of defensive secondary metabolites such as saponins to enhance chemical defense mechanisms, thereby adapting to environmental biotic and abiotic stresses.

The quantification of saikosaponins a, b2, c, and d (**Figures 3I–L**) revealed a decrease followed by an increase in content across BSC growth years. Saikosaponins a, c, and d were the most abundant in BSC1, followed by BSC3 and BSC2. Saikosaponin b2 content was the highest in BSC3, followed by BSC1 and BSC2. High-dose administration of saikosaponins a and d has been linked to cardiac toxicity (Wang et al., 2017), liver toxicity (Sun and Huang, 2010; Zhang F. et al., 2016; Li et al., 2017), and cognitive dysfunction (Lixing et al., 2018). Saikosaponin d undergoes conversion to saikosaponin b2 in gastric acid (Shimizu et al., 1985), with similar transformations observed after vinegar processing of *Radix Bupleuri* to increase saikosaponin b2 content (Li et al., 2015).

Slow release of saikosaponins a and d, combined with saikosaponin d2’s conversion to b2 could reduce liver toxicity, enhance liver-targeting effects, and improve hepatoprotective activity, achieving a “synergistic effect with reduced toxicity” (Zhao R. et al., 2010; Zhao et al., 2014; Wang et al., 2017; Shiu et al., 2020; Wang et al., 2022). Therefore, higher saikosaponin b2 content, along with balanced levels of saikosaponins a and d, may be a standard for assessing the efficacy and safety of *B. scorzonerifolium*. These saikosaponins are also key active components contributing to the antidepressant effects of *B. scorzonerifolium* (Wu et al., 2023). Among three BSC samples, only BSC3 was the most similar to BSW in the content of saikosaponins a, b2, c, and d, suggesting that BSC3 may possess antidepressant effects similar to those of BSW. The methodologies for determining total saponins and saikosaponins a, b2, c, and d are detailed in **Supplementary Tables 1** and **2**.

The volatile oil content showed no significant difference between BSC1 and BSC2 but increased significantly (*p* < 0.01) from BSC2 to BSC3. The volatile oil content in BSC3 was similar to that of BSW, ranging from 19% to 22% (**Figure 3H**). Volatile oils from *B. scorzonerifolium* exhibit antipyretic effects that are positively correlated with dosage (Wang et al., 2013). These findings suggest that, under equivalent dosages, BSC3 may exhibit superior antipyretic effects compared to BSC1 and BSC2, with efficacy most similar to that of BSW.

### 3.5 Metabolomics analysis

To further elucidate the metabolic differences among BSC1, BSC2, BSC3, and BSW, comprehensive metabolic profiling was conducted using LC-MS and GC-MS technologies.

#### 3.5.1 Metabolomics analysis based on LC-MS

**Figure 4:**
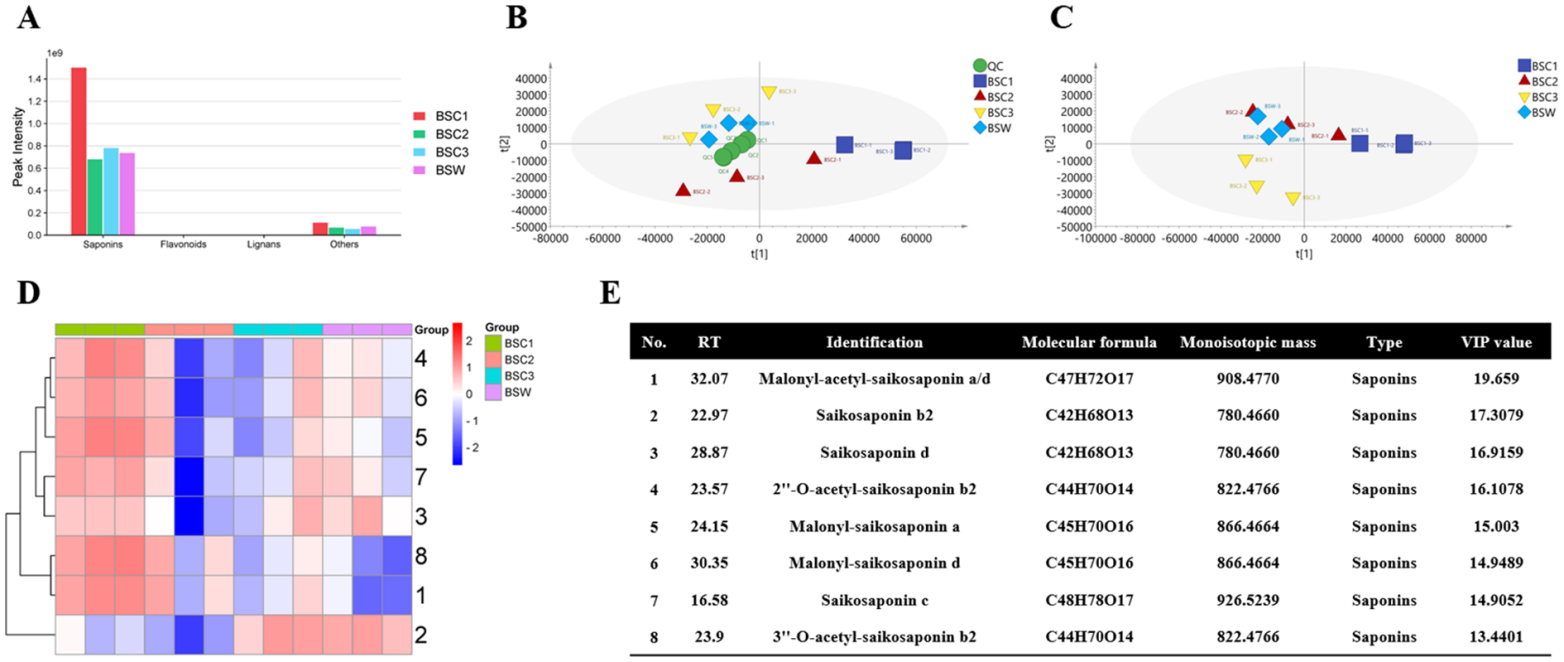
LC-MS-based metabolomic analysis of cultivated *Bupleurum scorzonerifolium* at 1-3 growth years (BSC1, BSC2, and BSC3) and wild *Bupleurum scorzonerifolium* (BSW). **(A)** Histogram of the relative content of various chemical components; **(B)** PCA plot; **(C)** PLS-DA plot; **(D)** Heatmap of the top eight DAMs ranked by VIP value; **(E)** Information table of the top eight DAMs ranked by VIP value.

Using LC-MS technology, 122 metabolites were identified, including saponins (99, 81.15%), flavonoids (4, 3.28%), lignans (3, 2.50%), and others (16, 13.11%). Although these compounds were detected in all samples, their relative abundances varied. Saponins exhibited the highest relative abundance (**Figure 4A**). The relative saponin content across the 1–3 years showed a trend of initial decrease followed by an increase, consistent with the total-saponins content measurements in Section 3.4.

To identify significantly differentially accumulated metabolites (DAMs), PCA and PLS-DA analyses were conducted. PCA showed well-clustered QC samples, indicating high instrument repeatability and reliable results. A clear separation was observed among the different groups, with BSC3 and BSW being closest to each other, both predominantly located in the second quadrant (**Figure 4B**). PLS-DA analysis further confirmed a distinct separation trend among the groups, with BSC1 being the furthest from the others (**Figure 4C**). Cross-validation with 200 repetitions and six-fold cross-validation indicated that the PLS-DA model was not overfitted (**Supplementary Figure 4A**).

The top eight compounds ranked by VIP scores were considered high-contribution DAMs (**Figure 4E**). Among these, all eight DAMs were saponins, and six showed a trend of initial decrease, followed by an increase across the 1–3 years, including the three key saikosaponins: saikosaponins b2, c, and d. Saikosaponins c and d showed the highest relative abundance in BSC1, followed by BSW, BSC3, and BSC2. Conversely, saikosaponin b2 exhibited the highest abundance in BSW and BSC3, followed by BSC1 and BSC2 (**Figure 4D**). These findings align with the quantitative analysis of saikosaponins b2, c, and d described in Section 3.4.

#### 3.5.2 Metabolomic analysis based on GC-MS

Using GC-MS technology, 98 metabolites were identified, including alkanes (39, 39.80%), aldehydes (17, 17.35%), fatty acids (10, 10.20%), alcohols (10, 10.20%), terpenes (7, 7.14%), esters (4, 4.08%), and other compounds (11, 11.22%). Although these metabolites were detected in all samples, their relative contents varied.

**Figure 5:**
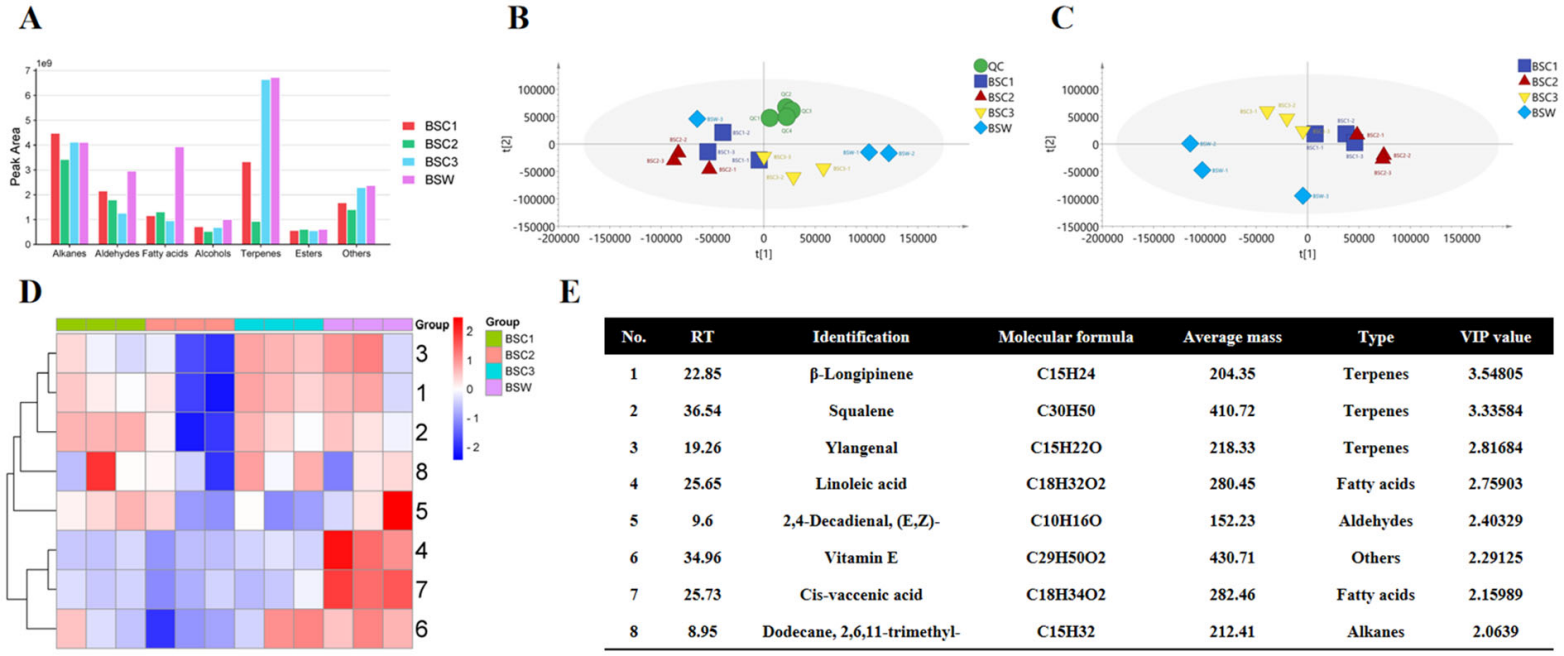
GC-MS-based metabolomic analysis of cultivated *Bupleurum scorzonerifolium* at 1-3 growth years (BSC1, BSC2, and BSC3) and wild *Bupleurum scorzonerifolium* (BSW). **(A)** Histogram of the relative content of various chemical components; **(B)** PCA plot; **(C)** PLS-DA plot; **(D)** Heatmap of the top eight DAMs ranked by VIP value; **(E)** Information table of the top eight DAMs ranked by VIP value.

As shown in **Figure 5A**, alkanes, terpenes, aldehydes, and fatty acids exhibited relatively high relative contents. Notably, the relative terpene content varied significantly among the BSC samples. Fatty acid content was significantly higher in BSW than in BSC, representing a key distinguishing feature of BSW.

To identify significant DAMs, PCA and PLS-DA analyses were conducted. PCA showed that all QC samples were well-clustered, indicating high reproducibility and reliable results (**Figure 5B**). PLS-DA analysis revealed distinct clustering trends among the groups, with BSC1 and BSC2 located in the right half of the plot, and BSW and BSC3 in the left half (**Figure 5C**). Cross-validation with 200 repetitions and six-fold cross-validation confirmed that the PLS-DA model was not overfitted (**Supplementary Figure 4B**).

The top eight DAMs ranked by VIP scores were identified as high-contribution metabolites (**Figure 5E**). Among these, seven DAMs showed a trend of initial decrease followed by an increase across the 1–3 years, including 3 terpenes, 2 fatty acids, 1 alkane, and 1 other compound (**Figure 5D**). The top three DAMs were all terpenes, including β-Longipinene, Ylangenal, and squalene, which was an important intermediate in the biosynthesis of saikosaponins (Sui et al., 2021). These terpenes contributed significantly to the differences in terpene content among BSC1, BSC2, and BSC3.

Additionally, BSW exhibited higher relative levels of two unsaturated fatty acids (compounds 4 and 7) than BSC. These unsaturated fatty acids were key contributors to the observed differences in fatty acid content between BSW and BSC.

Integrating the above results, it is evident that cultivated *B. scorzonerifolium* at 1–3 growth years exhibit significant differences in macroscopic traits, microstructures, cell wall component content, carbohydrate content, major bioactive compounds content, and metabolomic profiles, indicating that growth duration substantially influences its medicinal quality. Additionally, as one of the important material bases of the medicinal quality of *B. scorzonerifolium*, the content of saponins was affected by different environmental and cultivation factors. For example, saponin biosynthesis was enhanced under water-deficient or drought conditions (Zhu et al., 2009b; Zhang Y. et al., 2016). Excessive nitrogen and phosphorus fertilization reduces the total yield of saikosaponins a and d (Zhu et al., 2009a).

In this study, however, the growing environment and cultivation practices of all cultivated samples were consistent. Therefore, the observed quality variations are primarily attributed to growth year-dependent differential gene expression patterns. Notably, saponins and terpenoids were identified as the most significantly differentially accumulated bioactive metabolites among cultivated *B. scorzonerifolium* of different growth years. The molecular mechanisms underlying their differential accumulation deserved further exploration.

### 3.6 Transcriptome analysis

#### 3.6.1 Comparative transcriptome analysis

RNA-seq analysis was conducted for BSC1, BSC2, BSC3, and BSW. Approximately 39.85–51.13 million raw reads, 38.07–48.61 million clean reads, and 5.71–7.29 Gb of clean bases were obtained per sample. The Q20 and Q30 base percentages for all samples exceeded 98.2% and 94.8%, respectively, with GC content ranging from 41.19% to 42.78% (**Supplementary Table 3**). The mapping rate to the *B. scorzonerifolium* reference genome exceeded 82.44%.

Pearson correlation analysis showed that biological replicates in each group were well correlated (**Supplementary Figure 5A**). PCA results demonstrated clustering within groups and clear dispersion between groups, with BSW and BSC3 positioned in the left half of the PCA plot, while BSC1 and BSC2 were located in the right half (**Supplementary Figure 5B**).

#### 3.6.2 Metabolic pathway analysis of candidate genes involved in saponin and terpene biosynthesis

**Figure 6:**
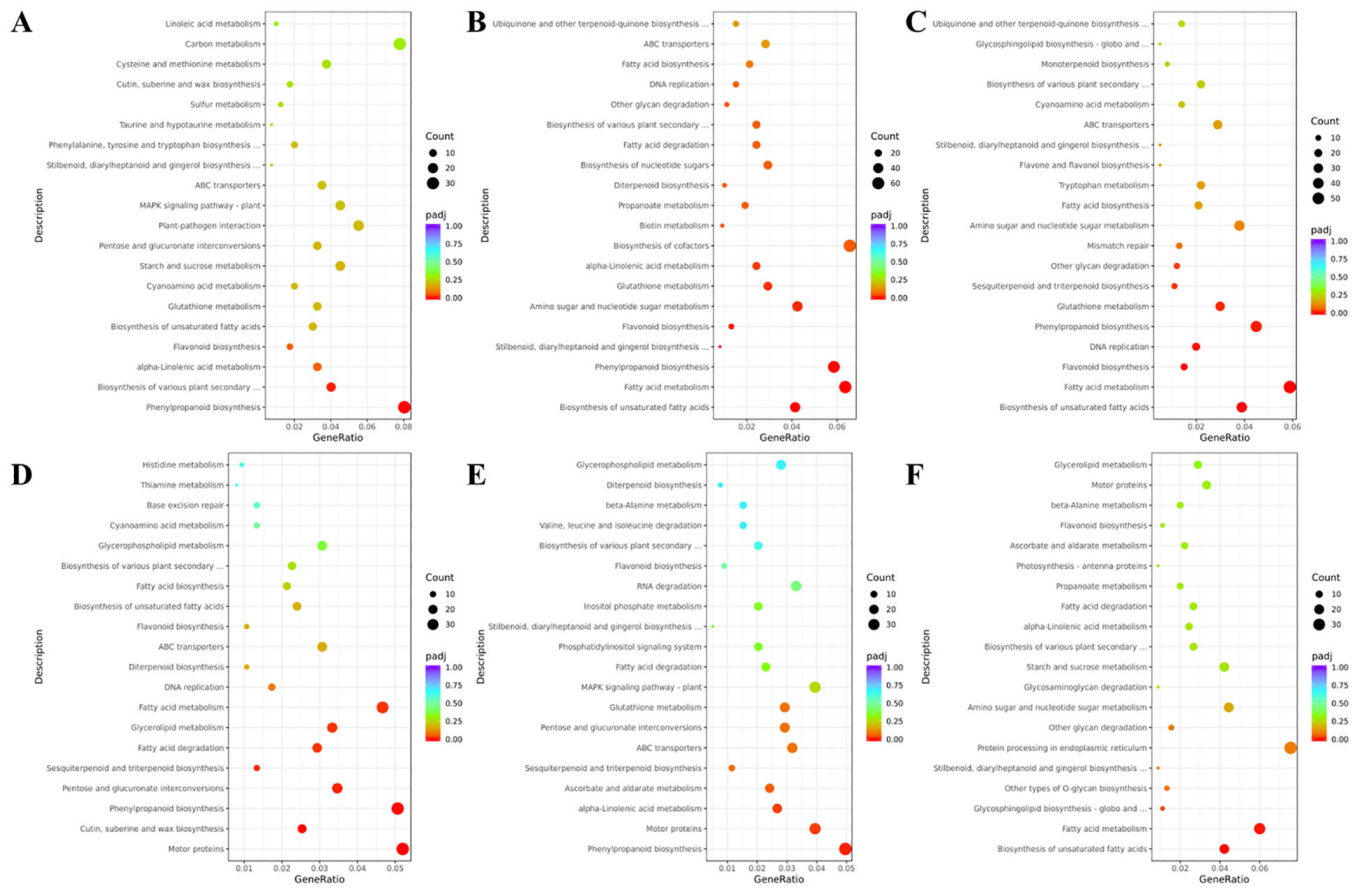
KEGG bubble plots of DEGs of cultivated *Bupleurum scorzonerifolium* at 1-3 growth years (BSC1, BSC2, and BSC3) and wild *Bupleurum scorzonerifolium* (BSW). **(A)** BSC2 vs. BSC1; **(B)** BSC3 vs. BSC2; **(C)** BSC3 vs. BSC1; **(D)** BSC1 vs. BSW; **(E)** BSC2 vs. BSW; **(F)** BSC3 vs. BSW. Those in the front and the back of the “vs.” are the treatment and the control groups, respectively.

To explore the molecular mechanisms underlying the differential accumulation of saponins and terpenes in BSC1, BSC2, BSC3, and BSW, DEG-Seq analysis was conducted, resulting in an overview of six groups of DEGs (**Supplementary Figure 6**). KEGG pathway annotation and enrichment analysis of these DEGs revealed significant enrichment in pathways, such as carbon metabolism, phenylpropanoid biosynthesis, fatty acid metabolism, biosynthesis of unsaturated fatty acids, and sesquiterpenoid and triterpenoid biosynthesis (*p* < 0.05) (**Figures 6A-F**). Among these pathways, 11 DEGS were annotated in the sesquiterpenoid and triterpenoid biosynthesis pathways (FPKM > 0). These genes encode seven enzymes: NAD+-dependent farnesol dehydrogenase (FDH), (-)-beta-caryophyllene synthase (CS), alpha-humulene synthase (HS), squalene synthase (SQS), squalene epoxidase (SE), camelliol C synthase (CAMS), and beta-amyrin synthase (β-AS). The first three enzymes are involved in the sesquiterpenoid biosynthesis pathway, while the latter four are related to the triterpenoid saponin biosynthesis pathway.

**Figure 7:**
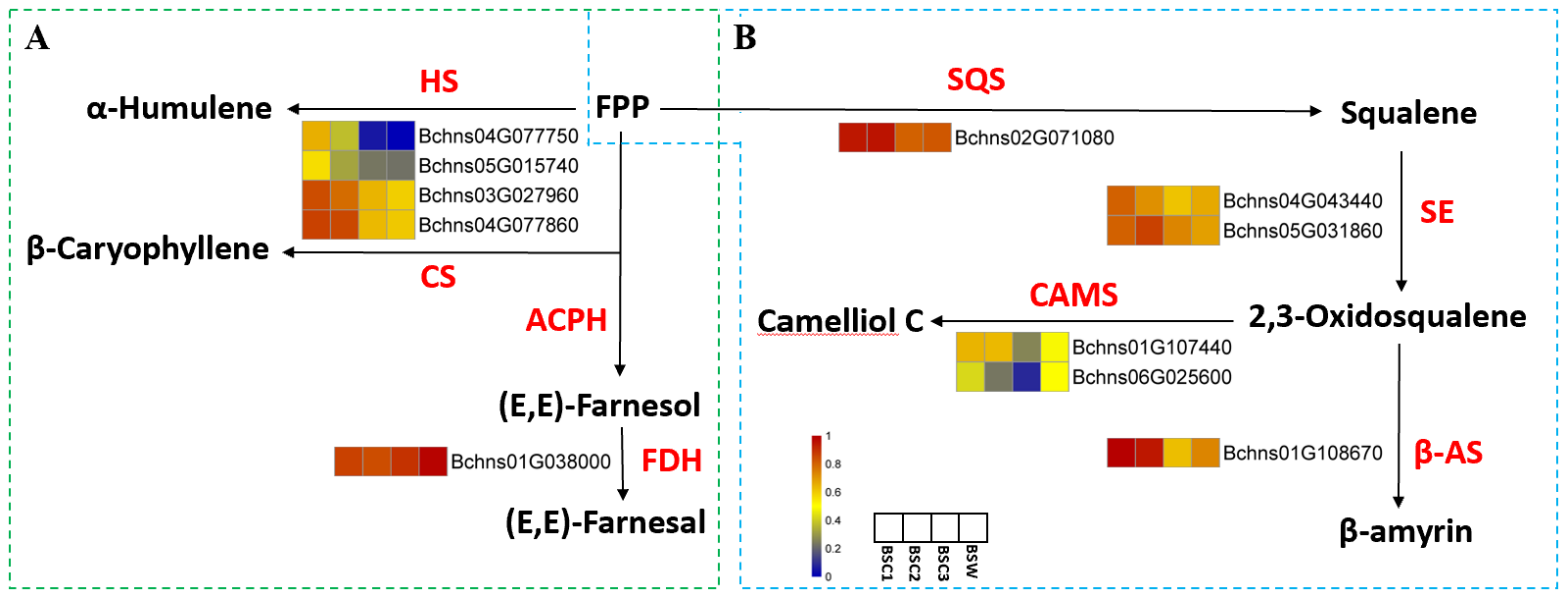
Pathway diagrams of DEGs annotated in sesquiterpene and triterpenoid biosynthesis in cultivated *Bupleurum scorzonerifolium* at 1-3 growth years (BSC1, BSC2, and BSC3) and wild *Bupleurum scorzonerifolium* (BSW). **(A)** Pathway diagram of sesquiterpene biosynthesis; **(B)** Pathway diagram of triterpenoid biosynthesis (second stage). Labels: HS, α-Humulene synthase; CS, (-)-β-caryophyllene synthase; ACPH Acetylpyruvate hydrolase; FDH, NAD+-dependent farnesol dehydrogenase; SQS, Squalene synthase; SE, Squalene epoxidase ; β-AS, β-Amyrin synthase; CAMS, Camelliol C synthase.

In the sesquiterpenoid biosynthesis pathway (**Figure 7A**), farnesyl diphosphatase catalyzes farnesyl pyrophosphate (FPP) conversion into (E,E)-farnesol, which is further converted to (E,E)-farnesal by FDH. The gene coding for this enzyme (*Bchns01G038000*) exhibited an initially increasing and then decreasing expression trend across BSC1 to BSC3, with the highest expression in BSW. This pattern aligns with sesquiterpene content variations, suggesting that *Bchns01G038000* may function as a key positive regulator. HS and CS also catalyze FPP conversion into α-humulene and β-caryophyllene, respectively. The genes encoding these enzymes (*Bchns04G077750*, *Bchns05G015740*, *Bchns03G027960*, and *Bchns04G077860*) showed progressively lower expression levels with increased cultivation years, with the lowest expression in BSW. To the best of our knowledge, this is the first report on the sesquiterpene biosynthesis pathway in *B. scorzonerifolium*, providing valuable directions for future studies.

The biosynthesis of saikosaponins involves three stages: precursor formation, scaffold construction, and post-modification. In the first stage, acetyl-CoA serves as the starting substrate and forms FPP through the mevalonate (MVA) pathway. In the second stage, SQS, SE, and β-AS catalyze FPP conversion into β-amyrin – a precursor of triterpene compounds. The third stage involves hydroxylation/oxidation, glycosylation, methylation, and β-amyrin acylation to produce structurally diverse saikosaponins (Sui et al., 2021).

Five DEGs were annotated in the second stage of saikosaponin biosynthesis (**Figure 7B**). SQS catalyzes FPP conversion into squalene. The gene encoding this enzyme (*Bchns02G071080*) exhibited an initially increasing and then decreasing expression pattern across BSC samples, with expression levels in BSW similar to those in BSC3. This trend, which contrasts with the expression levels of squalene, suggests that *Bchns02G071080* may function as a key negative regulator. Squalene is further converted by SE into 2,3-oxidosqualene. The two genes encoding SE (*Bchns04G043440* and *Bchns05G031860*) showed distinct trends; notably, *Bchns05G031860* exhibited a trend of first increasing and then decreasing, which contrasts with changes in saponin content, indicating it may also act as a negative regulator.

β-AS and CAMS subsequently catalyze 2,3-oxidosqualene conversion into β-amyrin and camelliol C, respectively. The expression levels of the genes encoding these enzymes (*Bchns01G108670* for β-AS, *Bchns01G107440* and *Bchns06G025600* for CAMS) showed a decreasing trend from BSC1 to BSC3.

In summary, *Bchns01G03800* appears to be a key positive regulator of sesquiterpene content influenced by cultivation years. *Bchns02G071080* and *Bchns05G031860*, respectively encoding SQS and SE, were identified as potential negative regulators in triterpenoid saponin biosynthesis (Zhao C. et al., 2010).

## 4 Conclusion

This study is the first to investigate the quality differences between BSC at 1–3 years of cultivation and BSW while exploring the molecular mechanisms underlying these differences. The results demonstrate that during the growth period from 1 to 3 years, BSC undergoes gradual increases in weight, upper root diameter, volatile oil content, and number and size of oil ducts and cavities. Meanwhile, the PXR and the contents of total-saponins, as well as saikosaponins a, b2, c, and d, initially decrease and then increase. Saponins and terpenes were identified as the main contributors to the metabolic differences in BSC across 1–3 years of growth. The genes *Bchns02G071080* and *Bchns05G031860* (associated with saponins) and *Bchns01G03800* (associated with terpenes) emerged as potential key regulatory genes influencing the accumulation of these compounds during cultivation. In conclusion, based on trait characteristics, microscopic structure, chemical composition, metabolic profiles, and transcriptomic data, BSC3 most closely resembles BSW. From both quality and economic perspectives, it is recommended to harvest *B. scorzonerifolium* after 3 years of cultivation. However, this study is limited by the absence of cultivated samples older than 3 years. Therefore, whether BSC older than 3 years may surpass BSC3 in its resemblance to BSW across all aspects remains unanswered. Future research should include higher-age cultivated samples to further validate these findings and refine recommendations for the optimal cultivation period.

## Data Availability

The original contributions presented in the study are included in the article/**Supplementary Material**. Further inquiries can be directed to the corresponding author/s.
