# Supplementary material for: Effects of growth years on the quality of cultivated Bupleurum scorzonerifolium roots, with wild Bupleurum scorzonerifolium roots as a reference
Source: Front Plant Sci. 2025 Mar 31;16:1552429. doi: 10.3389/fpls.2025.1552429 (PMC11994674; doi:10.3389/fpls.2025.1552429)
Supplement: Supplementary file 1 [file DataSheet1.docx]

***Supplementary Material***

**Figures and Tables**

**Supplementary Figure 1.** Fine hair-like dead leaf fibers images of cultivated *Bupleurum scorzonerifolium* at 1-3 growth years (BSC1, BSC2, and BSC3) and wild *Bupleurum scorzonerifolium* (BSW). (**A**) BSC1; (**B**) BSC2; (**C**) BSC3; (**D**) BSW. Inside the red dashed box were fine hair-like dead leaf fibers.

**Supplementary Figure 2.** Annular striations images of cultivated *Bupleurum scorzonerifolium* at 1-3 growth years (BSC1, BSC2, and BSC3) and wild *Bupleurum scorzonerifolium* (BSW). (**A**) BSC1; (**B**) BSC2; (**C**) BSC3; (**D**) BSW. Labels: a, no or inconspicuous annular striations; b, annular striations. The red dashed box showed the area where the annular striations were located.

**Supplementary Figure 3.** Weight (**A**), Length (**B**), Upper diameter (**C**) , Middle diameter (**D**) and Lower diameter (**E**) of cultivated *Bupleurum scorzonerifolium* at 1-3 growth years (BSC1, BSC2 and BSC3) and wild *Bupleurum scorzonerifolium* (BSW). *, *p*<0.05; **, *p*<0.01; ***, *p*<0.001; ****, *p*<0.0001.

**Supplementary Figure 4.** 200-fold cross-validation plots of the PLS-DA model for LC-MS data (**A**) and GC-MS data (**B**) of cultivated *Bupleurum scorzonerifolium* at 1-3 growth years (BSC1, BSC2 and BSC3) and wild *Bupleurum scorzonerifolium* (BSW).

**Supplementary Figure 5.** Transcriptomic analysis of cultivated *Bupleurum scorzonerifolium* at 1-3 growth years (BSC1, BSC2, and BSC3) and wild *Bupleurum scorzonerifolium* (BSW). (**A**) Correlation analysis plot; (**B**) PCA plot.

**Supplementary Figure 6.** Volcano plots of DEGs of cultivated *Bupleurum scorzonerifolium* at 1-3 growth years (BSC1, BSC2, and BSC3) and wild *Bupleurum scorzonerifolium* (BSW). (**A**) BSC2 vs. BSC1; (**B**) BSC3 vs. BSC2; (**C**) BSC3 vs. BSC1; (**D**) BSC1 vs. BSW; (**E**) BSC2 vs. BSW; (**F**) BSC3 vs. BSW. Those in the front and the back of the “vs.” are the treatment and the control groups, respectively.

**Supplementary Table** **1.** Calibration curves, *R^2^* and Linear Range of water-soluble polysaccharide and total-saponins.

**Supplementary Table** **2.** Calibration curves, precision, repeatability and stability of four active compounds.

**Supplementary Table** **3.** Summary of transcriptome data of cultivated *Bupleurum scorzonerifolium* at 1-3 growth years (BSC1, BSC2, and BSC3) and wild *Bupleurum scorzonerifolium* (BSW).


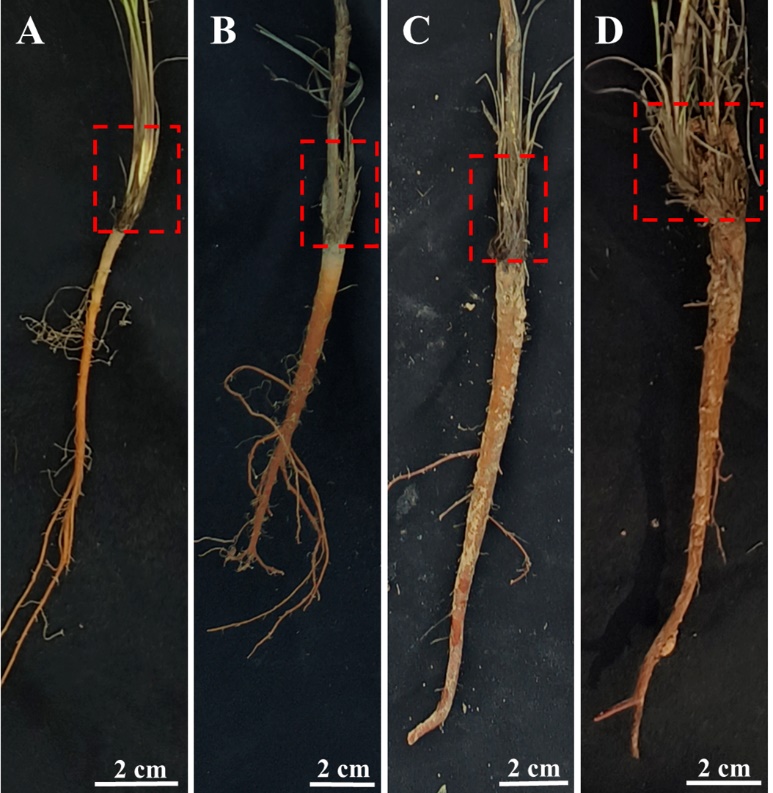


**Supplementary Figure 1.** Fine hair-like dead leaf fibers images of cultivated *Bupleurum scorzonerifolium* at 1-3 growth years (BSC1, BSC2, and BSC3) and wild *Bupleurum scorzonerifolium* (BSW). (**A**) BSC1; (**B**) BSC2; (**C**) BSC3; (**D**) BSW. Inside the red dashed box were fine hair-like dead leaf fibers.


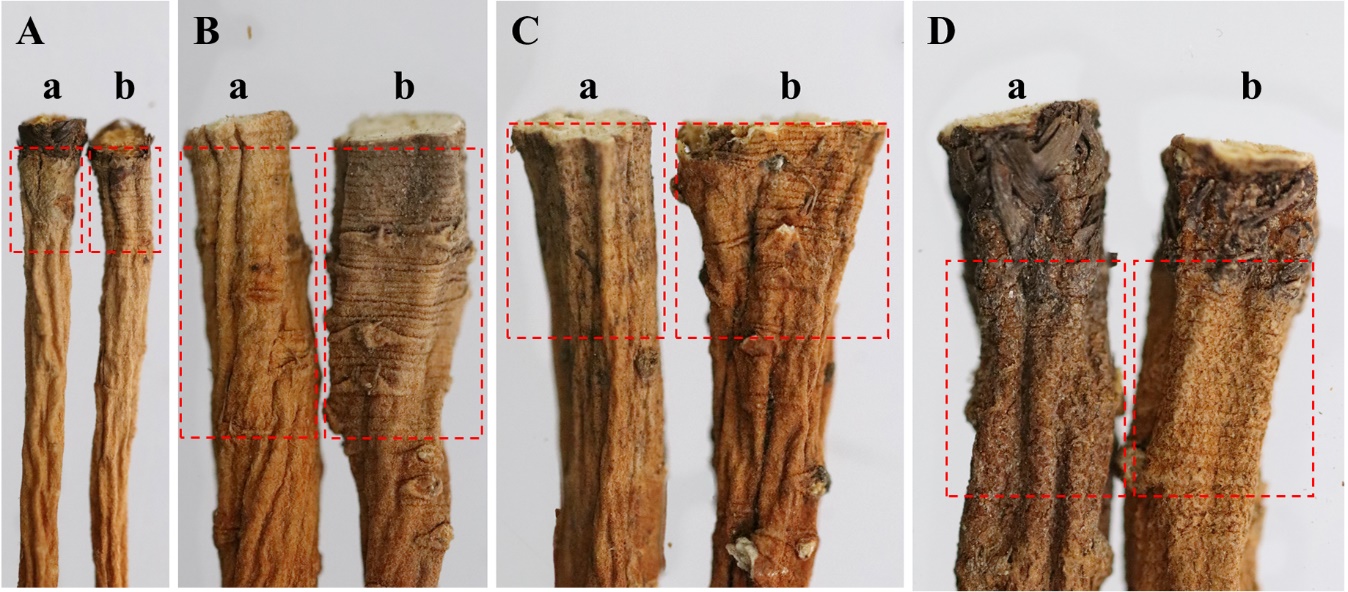


**Supplementary Figure 2.** Annular striations images of cultivated *Bupleurum scorzonerifolium* at 1-3 growth years (BSC1, BSC2, and BSC3) and wild *Bupleurum scorzonerifolium* (BSW). (**A**) BSC1; (**B**) BSC2; (**C**) BSC3; (**D**) BSW. Labels: a, no or inconspicuous annular striations; b, annular striations. The red dashed box showed the area where the annular striations were located.


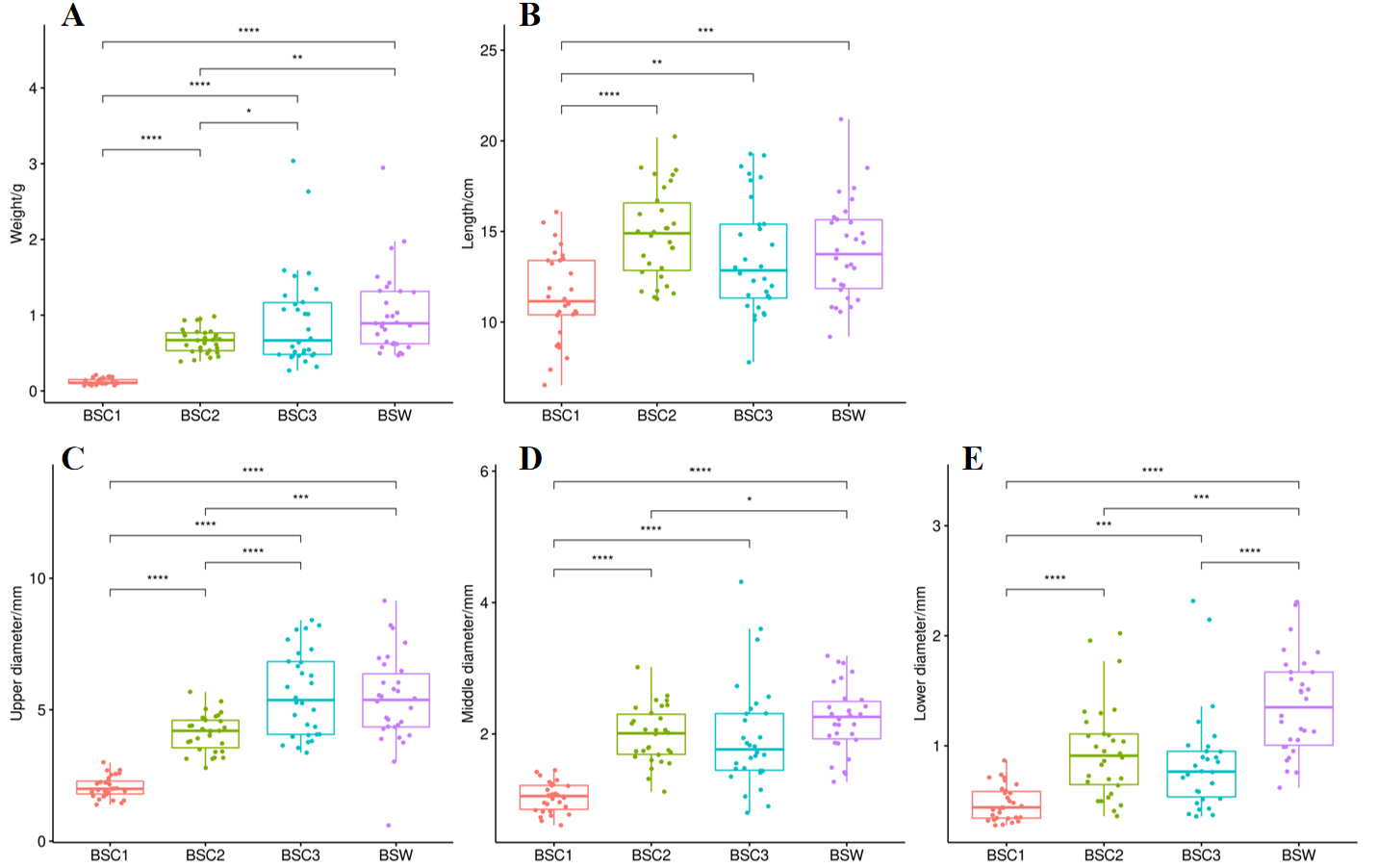


**Supplementary Figure 3.** Weight (**A**), Length (**B**), Upper diameter (**C**) , Middle diameter (**D**) and Lower diameter (**E**) of cultivated *Bupleurum scorzonerifolium* at 1-3 growth years (BSC1, BSC2 and BSC3) and wild *Bupleurum scorzonerifolium* (BSW). *, *p*<0.05; **, *p*<0.01; ***, *p*<0.001; ****, *p*<0.0001.


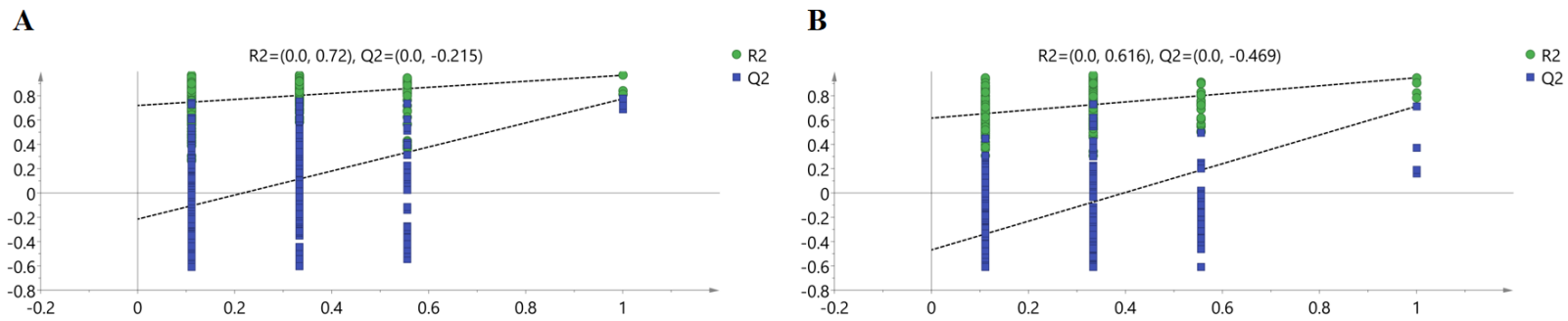


**Supplementary Figure 4.** 200-fold cross-validation plots of the PLS-DA model for LC-MS data (**A**) and GC-MS data (**B**) of cultivated *Bupleurum scorzonerifolium* at 1-3 growth years (BSC1, BSC2 and BSC3) and wild *Bupleurum scorzonerifolium* (BSW).


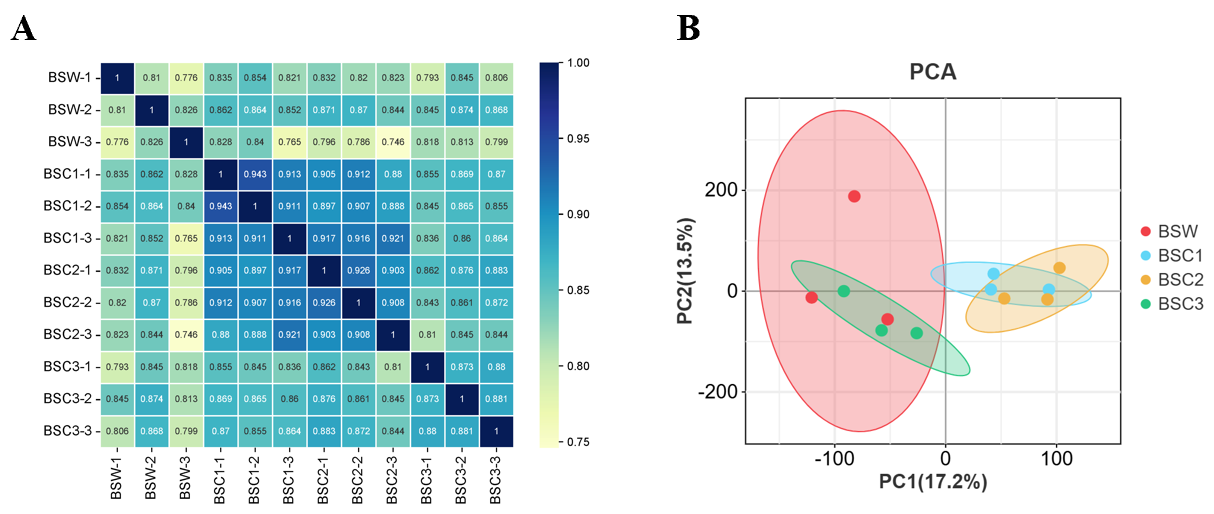


**Supplementary Figure 5.** Transcriptomic analysis of cultivated *Bupleurum scorzonerifolium* at 1-3 growth years (BSC1, BSC2, and BSC3) and wild *Bupleurum scorzonerifolium* (BSW). (A) Correlation analysis plot; (B) PCA plot.


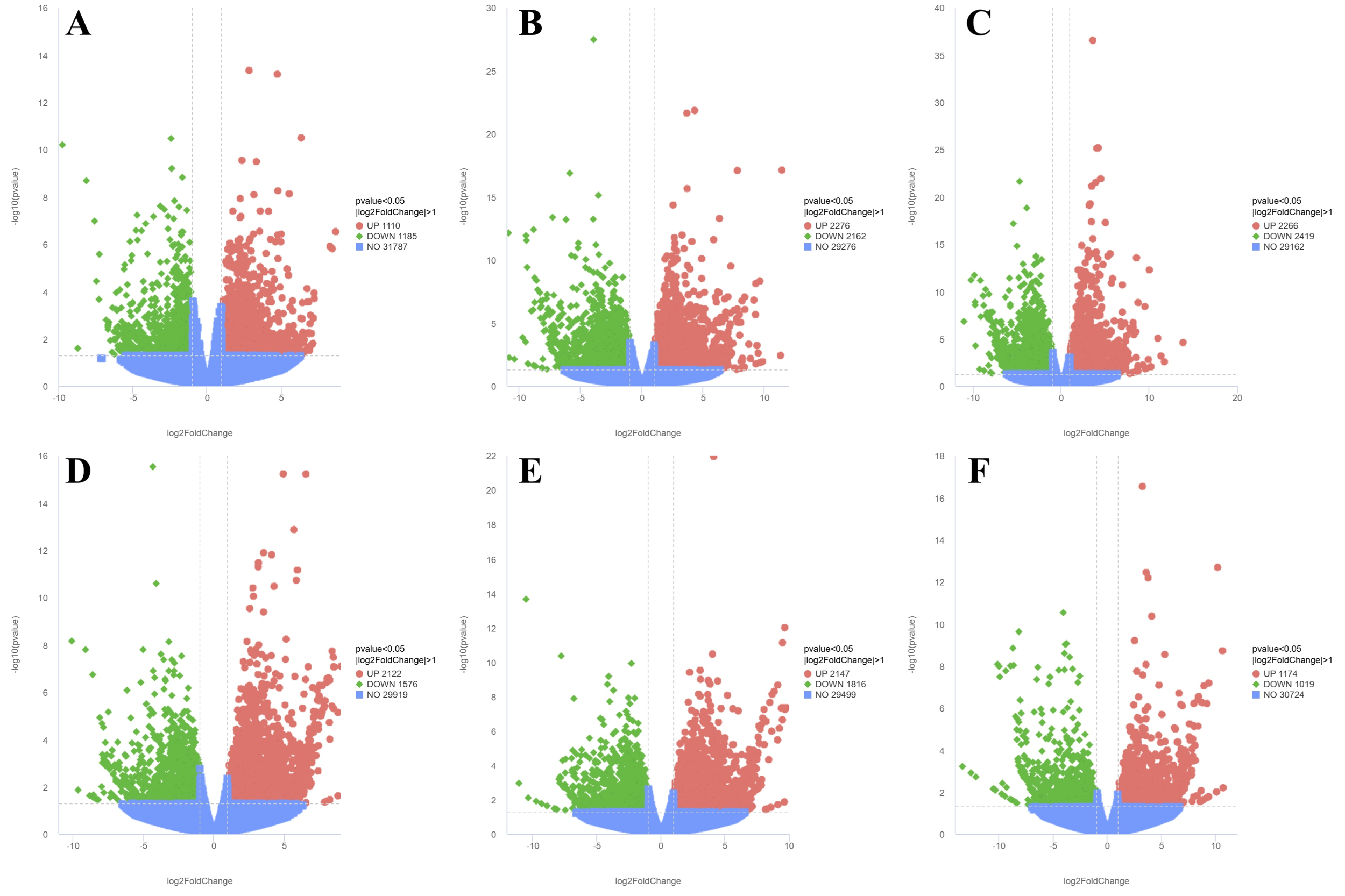


**Supplementary Figure 6.** Volcano plots of DEGs of cultivated *Bupleurum scorzonerifolium* at 1-3 growth years (BSC1, BSC2, and BSC3) and wild *Bupleurum scorzonerifolium* (BSW). (**A**) BSC2 vs. BSC1; (**B**) BSC3 vs. BSC2; (**C**) BSC3 vs. BSC1; (**D**) BSC1 vs. BSW; (**E**) BSC2 vs. BSW; (**F**) BSC3 vs. BSW. Those in the front and the back of the “vs.” are the treatment and the control groups, respectively.

**Supplementary Table** **1.** Calibration curves, *R^2^* and Linear Range of water-soluble polysaccharide and total-saponins. (x: absorbance; y: concentration.)

|  | Compound | Calibration curves | *R*^2^ | Linear Range (mg/mL) |
| --- | --- | --- | --- | --- |
| Water-soluble polysaccharide | Sucrose | y = 0.2279 x - 0.0227 | 0.9967 | 0.015625-1 |
| Total-Saponins | Saikosaponin a | y = 5.3214 x - 0.0562 | 0.9948 | 0.25-6 |

**Supplementary Table** **2.** Calibration curves, precision, repeatability and stability of saikosaponins a, b2, c, and d. (x: absorbance; y: concentration.)

| Compound | Calibration curves | *R*^2^ | Linear Range  (mg/mL) | Precision  (RSD, n=6) | Repeatability (RSD, n=6) | Stability  (RSD, n=6) |
| --- | --- | --- | --- | --- | --- | --- |
| Saikosaponin a | log_2_y = 0.7371 log_2_x - 14.853 | 0.9994 | 0.033125-3.18 | 0.82% | 4.31% | 3.60% |
| Saikosaponin b2 | log_2_y = 0.6777 log_2_x - 14.108 | 0.9991 | 0.01875-1.8 | 3.53% | 3.82% | 3.22% |
| Saikosaponin c | log_2_y = 0.7086 log_2_x - 14.629 | 0.9994 | 0.03625-3.48 | 1.00% | 4.47% | 4.51% |
| Saikosaponin d | log_2_y = 0.748 log_2_x - 14.71 | 0.9991 | 0.0334375-3.21 | 1.66% | 4.00% | 3.99% |

**Supplementary Table** **3.** Summary of transcriptome data of cultivated *Bupleurum scorzonerifolium* at 1-3 growth years (BSC1, BSC2, and BSC3) and wild *Bupleurum scorzonerifolium* (BSW).

| Sample | Raw Reads | Clean Reads | Clean bases | Error rate  (%) | Q20 (%) | Q30 (%) | GC content (%) | Mapped ratio |
| --- | --- | --- | --- | --- | --- | --- | --- | --- |
| BSC1-1 | 51135104 | 46940880 | 7.04G | 0.02 | 98.56 | 95.49 | 42 | 39984916(85.18%) |
| BSC1-2 | 48419772 | 44763144 | 6.71G | 0.02 | 98.57 | 95.38 | 42.54 | 38492619(85.99%) |
| BSC1-3 | 44633272 | 44609618 | 6.69G | 0.02 | 98.57 | 95.43 | 42.4 | 38480662(86.26%) |
| BSC2-1 | 43287042 | 40885942 | 6.13G | 0.02 | 98.63 | 95.67 | 42.47 | 34927053(85.43%) |
| BSC2-2 | 39855338 | 38070494 | 5.71G | 0.02 | 98.65 | 95.58 | 42.58 | 32920040(86.47%) |
| BSC2-3 | 41172600 | 39025472 | 5.85G | 0.02 | 98.32 | 94.87 | 42.6 | 33323431(85.39%) |
| BSC3-1 | 41331580 | 39183044 | 5.88G | 0.02 | 98.4 | 95.17 | 42.07 | 32632586(83.28%) |
| BSC3-2 | 49515918 | 48615316 | 7.29G | 0.02 | 98.61 | 95.51 | 41.44 | 40672905(83.66%) |
| BSC3-3 | 42030490 | 39754700 | 5.96G | 0.02 | 98.29 | 94.83 | 41.65 | 33337474(83.86%) |
| BSW-1 | 45926182 | 43266642 | 6.49G | 0.02 | 98.55 | 95.38 | 42.78 | 36575661(84.54%) |
| BSW-2 | 41904320 | 38685590 | 5.8G | 0.02 | 98.38 | 95.22 | 41.19 | 31890710(82.44%) |
| BSW-3 | 42021146 | 38638072 | 5.8G | 0.02 | 98.53 | 95.48 | 42.09 | 32331916(83.68%) |
